# Pharmacist-led Si-care (schizophrenia care) model to improve medication adherence and symptom management in schizophrenia

**DOI:** 10.1016/j.rcsop.2024.100544

**Published:** 2024-11-22

**Authors:** Noor Cahaya, Susi Ari Kristina, Anna Wahyuni Widayanti, James A. Green

**Affiliations:** a Doctoral Program in Pharmacy, Faculty of Pharmacy, Universitas Gadjah Mada, Yogyakarta, Indonesia; b Department of Pharmacy, Faculty of Mathematics and Science, Universitas Lambung Mangkurat, Indonesia; c Department of Pharmaceutics, Faculty of Pharmacy, Universitas Gadjah Mada, Yogyakarta, Indonesia; d School of Allied Health and Physical Activity for Health, Health Research Institute (HRI), University of Limerick, Limerick, Ireland

**Keywords:** Adherence, Antipsychotic, PANSS, Pharmacist-led, Pill count, Schizophrenia

## Abstract

**Introduction:**

Schizophrenia is a chronic mental disorder that requires long-term treatment, particularly antipsychotic medications. However, medication adherence among patients with schizophrenia is often suboptimal, leading to symptom relapse and poor outcomes. The Si-Care (Schizophrenia Care) program was developed as a pharmacist-led home intervention to improve medication adherence and support symptom control in patients with schizophrenia. This study aimed to evaluate the effectiveness of the Si-Care intervention in improving medication adherence and maintaining stability of symptoms among schizophrenia patients.

**Methods:**

A quasi-experimental study was conducted in three community health centers or Puskesmas in Banjarmasin, Indonesia. A total of 57 participants were recruited according to the sampling criteria. The Si-Care intervention consisted of seven home visits by trained pharmacists over four months, providing education, counseling, and medication monitoring. Adherence to medication was evaluated using the pill count method and severity of symptoms was measured using the Positive and Negative Syndrome Scale (PANSS) pre- and post-intervention. Data were analyzed using the Friedman test for adherence and the Wilcoxon test for PANSS scores.

**Results:**

Mean medication adherence improved significantly from 77.38 % ± 25.85 at baseline (T0) to 97.57 % ± 11.09 at the final visit (T4) (*p* = 0.000). However, the decrease in PANSS scores from 38.03 ± 9.14 to 37.81 ± 9.15 was not statistically significant (*p* = 0.089). Despite the lack of significant change in PANSS scores, symptoms remained stable throughout the intervention, suggesting effective symptom management.

**Conclusions:**

The Si-Care intervention significantly improved medication adherence among people with schizophrenia, contributing to the maintenance of stable symptoms. Pharmacist-led home interventions provide valuable support to address adherence challenges and should be considered a critical component in schizophrenia care. Future studies should consider a more rigorous design, a larger sample size, and longer follow-up to better evaluate the sustainability, scalability, and applicability of the intervention in diverse healthcare settings.

## Highlights

• The Si-Care program is a pharmacist-led intervention featuring home visits, education, and medication monitoring for people with schizoprenia.
• The Si-Care intervention successfully improved medication adherence among people with schizophrenia, contributing to symptom stability.
• The intervention suggests that pharmacists can be pivotal in managing schizophrenia in community settings.

## 1 Introduction

Schizophrenia is a significant mental disorder marked by psychotic symptoms such as hallucinations and delusions.^1^ It impacts around 24 million individuals, which is approximately 1 in 300 people (0.32 %) globally, and necessitates long-term treatment involving medications and psychosocial therapies.^2^ Nevertheless, ensuring patients' adherence to prescribed medication poses a significant challenge in managing the condition.^3^^,^^4^

Medication adherence in schizophrenia is a major problem in the management of this disease.^3^^,^^5^ Side effects, such as sedation and extrapyramidal symptoms, are particularly problematic, often leading patients to stop taking their medications or to use them inconsistently .^6^^,^^7^ Moreover, people with schizophrenia frequently experience poor insight into their condition, making it challenging for them to recognize the need for continued treatment.^8^ Studies showed that medication non-adherence among people with schizophrenia ranges between 40 % and 60 %,^9^^,^^10^ which is caused by various factors such as drug side effects, lack of education about the importance of therapy, social stigma, and limited support from the health care system.^3^^,^^10^^,^^11^ These factors contribute to high rates of relapse and hospitalization, worsening long-term prognosis, and placing a significant burden on both patients and healthcare systems.^3^

Given the critical need to improve medication adherence in people with schizophrenia, pharmacist-led interventions have gained attention as an effective strategy. Pharmacists play a key role in managing medication regimens, educating patients and their families, addressing side effects, and providing ongoing support.^12^^,^^13^ Several studies have shown that when pharmacists are integrated into mental health care teams, they can significantly improve medication adherence, cognitive functions, reducing drug-related problems (DRPs), and overall treatment outcomes in people with schizophrenia.^14^^,^^15^

The pharmacist-led Si-Care intervention was developed with the aim of providing a more holistic approach to support adherence to medication in people with schizophrenia. Unlike other interventions that psychiatrists, psychologists or nurses typically lead,^16^^,^^17^ Si-Care is a mixed intervention that combines community pharmacist-led care, home visits, education, counseling, support, motivation and medication monitoring to ensure patients adhere to their medication regimen, filling a gap in more personalised and accessible care for schizophrenia. A systematic review and meta-analysis study showed that mixed interventions combining elements of education, motivational interviewing, and self-management of medications had a positive effect on patient adherence.^18^ Therefore, Si-Care is a home care program that incorporates pharmacists as a critical component of the mental health care team, specifically for people with schizophrenia; By addressing common barriers to adherence, such as side effects and lack of knowledge about the illness, the program seeks to improve medication adherence and symptom management in people with schizophrenia, particularly outpatients.

The Si-Care program is based in a community pharmacy, highlighting the differences between this program and other interventions from different studies.^19^, ^20^, ^21^ The program aims to help patients and their families better understand schizophrenia and the importance of medication therapy to address barriers to medication adherence and provide ongoing support. Therefore, this study aims to evaluate pharmacist interventions in the Si-care program during a study focusing on improving adherence to antipsychotic medications and symptom control in outpatients with schizophrenia.

## 2 Methods

### 2.1 Study design

A quasi-experimental pre and post, without control, study, conducted at Community Health Center or Puskesmas, in Banjarmasin, South Kalimantan Province.

### 2.2 Participants

Participants in this study were recruited from three Puskesmas (Puskesmas Pekauman, Alalak Tengah and Sungai Jingah) in Banjarmasin City, South Kalimantan Province. A total of 107 schizophrenia outpatients were selected from Puskesmas based on inclusion and exclusion criteria to participate in the study. The researcher established specific inclusion and exclusion criteria for the participants. A psychiatrist assisted the researcher in identifying potential participants who met the research criteria.

The inclusion criteria were as follows: the outpatient had a psychiatrist diagnosis of schizophrenia based on the criteria of the Diagnostic and Statistical Manual of Mental Disorders *(*DSM-V), was 18 years of age or older, used oral antipsychotics (typical or atypical), could comprehend information and respond to questions in Indonesian or local ethnic language, and was willing to give informed consent for the study (patient and the caregivers).

Exclusion criteria included individuals with organic brain disorders or mental retardation and severe comorbid conditions (such as drug dependence or chronic illness or infectious disease), and patients have changed their address, which may make it difficult for them to visit.

**Fig. 1:**
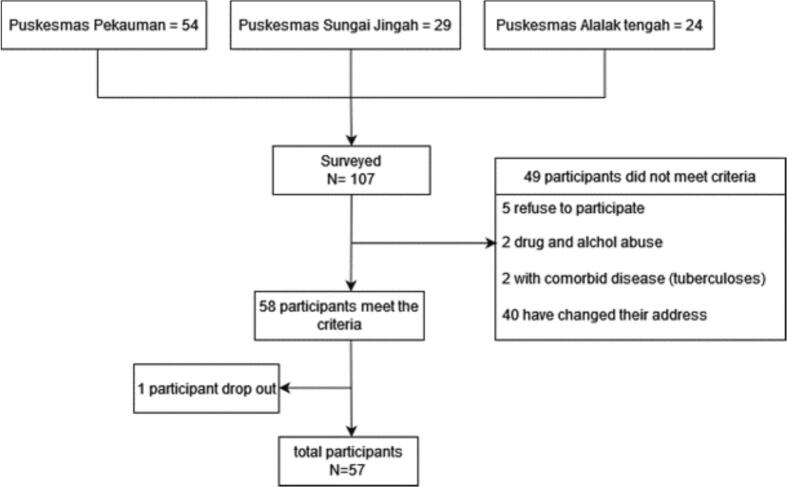
Participants Enrollment.

After selecting the participants who met the criteria, the researcher (first author) explained the study to the patient and his/her family. Fifty-seven participants agreed to participate in this study. Fig. 1 describes the recruitment of study participants.

### 2.3 Study setting

In the process of research license, Dinas Kesehatan Kota Banjarmasin recommended three Puskesmas or community health centres for potential sampling locations: Puskesmas Pekauman, Puskesmas Sungai Jingah, dan Puskesmas Alalak Tengah in Banjarmasin, South Borneo province, Indonesia. These Puskesmas were selected based on the prevalence of cases of mental disorders in Banjarmasin City. The Si-Care intervention is conducted in community settings, specifically in the homes of patients.

### 2.4 Interventions

The Si-Care (Schizophrenia Care) intervention is a pharmacy-based home care program. It includes education, counseling, monitoring, and evaluation of the therapy provided by pharmacists in the community. The aim is to increase knowledge and adherence to medications for people with schizophrenia.

The pharmacists who provided the intervention were community pharmacists who had received three days of previous training. They receive specialized training through workshops aimed at improving their ability to provide effective interventions to patients with schizophrenia. This training occurs before beginning home care visits. It includes intensive sessions focused on understanding schizophrenia and its treatment, improving communication skills, and motivational interviewing (MI) techniques, which are integral to the intervention. The aim was to provide comprehensive training to pharmacists, thus enhancing their theoretical knowledge and practical proficiency to provide effective counseling to people with schizophrenia. Training also includes role-plays and practical simulations, allowing pharmacists to practice their skills and receive feedback to improve their counseling techniques. The pharmacists were trained by psychiatrists, pharmacologists, and psychologists. Pharmacists are also measured for their knowledge at the beginning and end of the training, and a psychologist assesses their counseling skills. Based on the assessment results, all pharmacists who participated in this program's training were declared eligible.

In addition, pharmacists use booklets as educational media for patients and their families. The description of the material provided in the booklet is explained in Table 1.

**Table 1:** Description of the Booklet for Participants.

Booklet number	Contents	Schedule	Goal
1	Schizophrenia, signs and symptoms, and causes of schizophrenia.	2nd visit	To understand the nature of schizophrenia and its symptoms, understanding the causes of schizophrenia is necessary.
2	Antipsychotics, side effect and interaction, and withdrawal symptoms.	3rd visit	To understand the treatment of schizophrenia.
3	Medication adherence and non-adherence, consequences of non-adherence, and strategies to improve medication adherence.	4th visit	Understanding medication adherence and the impact of medication nonadherence
4	The role of the family in treatment, sign and symptoms of recurrence, and emergency management	5th visit	To improve participation and supports of family or caregivers in the treatment of schizophrenia

### 2.5 Procedure

The intervention was carried out at the participants' homes by a pharmacist. Each pharmacist visited patients' homes seven times over four months, each visit lasting an estimated 40–50 min (see Table 2). At the beginning of the visit, the pharmacist explained the Si-Care program and obtained informed consent at the first visit.

**Table 2:** Description of Visits' Activities.

Schedule	Description
1st visit	Introduces and explores patients' medication problems while measuring adherence and PANSS score, which are considered pre-intervention data (T0).
2nd visit	Two weeks after the first visit, the pharmacist visits the patient's home involved psychoeducation using booklet 1
3rd visit	Two weeks after the second visit, the pharmacist visits the patient's home involved psychoeducation using booklet 2, and adherence is measured in the first month (T1).
4th visit	Two weeks after the third visit, the pharmacist visits the patient's home involved psychoeducation using booklet 3.
5th visit	Two weeks after the fourth visit, the pharmacist visits the patient's home involved psychoeducation using booklet 4, and adherence is measured in the second month (T2).
6th visit	4 weeks after the fifth visit, the pharmacist visits only to monitor the patient's condition and measure adherence in the third month (T3).
7th visit	4 weeks after the sixth visit, the pharmacist visited to monitor the patient's condition, measure adherence (T4) in the fourth month and PANSS score post intervention

### 2.6 Ethics approval

Ethical clearance (EC) was granted by the Medical and Health Research Ethics Committee (MHREC) of the Faculty of Medicine, Public Health, and Nursing at Universitas Gadjah Mada (EC approval No. KE/FK/1210/EC/2023). A copy of the ethical clearance letter was provided to the administration for research approval from the Badan Kesatuan Bangsa dan Politik Kota Banjarmasin and Dinas Kesehatan Kota Banjarmasin, which are the study sites. All study participants provided their consent prior to data collection. Each participant, as well as the families or caregivers of schizophrenia patients, gave their informed, voluntary, written, and signed consent. Participants were also informed of their right to withdraw from the study at any time, and confidentiality of their information was guaranteed.

### 2.7 Data collection

The primary outcome measures with respect to the Si-Care intervention were reduced or controlled symptoms and increased patient medication adherence at the beginning and end of the intervention.

PANSS score data were evaluated before and after intervention with the help of a psychiatrist. PANSS, a symptom-based rating scale, is a standard tool that should be widely used in clinical diagnosis of schizophrenia and has good realiability and validity.^22^

The method of assessing medication adherence involves the indirect pill count method, which measures the compliance of the respondent by calculating the remaining medication. Total pills minus remaining pills divided by pills to be taken, multiplied by 100 %.^23^ For example, if a patient was supposed to take 30 pills in a month and only 20 pills are left, adherence can be calculated as (30−20) / 30 = 33 % adherence. The data from these counts are then analyzed to determine the level of adherence of patients to their antipsychotic medication.

The pill-counting method widely used and simple. It is considered an objective measure because the data obtained comes from direct observation or physical counting of the number of pills present rather than from the patient's memory or perception of how well they are following their medication regimen.^24^

Each patient is provided with a treatment control card that includes important information such as the name of the drug, dosage form, frequency, dosage strength, quantity dispensed, and the dispensing date. Patients must bring this card with them for their upcoming check-ups.

To ensure accuracy, the researcher cross-checks the information on the treatment control card with the drug label to verify that the quantity of medicine taken matches the prescribed amount.

### 2.8 Data analysis

The PANSS score and medication adherence were evaluated for normality, and then a non-parametric test was performed using a Wilcoxon test for the PANSS score and the Friedman test to analyze the significance of medication adherence.

## 3 Results

### 3.1 Participants characteristics

A total of 57 participants participated in the Si-Care intervention. The average age of the respondents was 40.42 years, 57.9 % of whom were male, and 52.6 % were unmarried. Most of the participants were unemployed and the number of those employed was less than 20 %. Furthermore, most of the participants have a low level of education and were diagnosed with undifferentiated schizophrenia (F20.3). The baseline characteristics of the participants are briefly described in Table 3.

**Table 3:** Baseline Characteristics.

Characteristics	No. of respondents (*N* = 57)	Percentage (%)
Diagnosis		
F20.0	8	14
F20.3	49	86
Age (mean ± SD)	40.42 ± 10.60	
19–39	24	42.11
40–59	30	52.63
≥60	3	5.26
Education level		
Primary school dropout	18	31.6
Primary school graduate	12	21.1
Junior high school graduate	12	21.1
High school graduate	15	26.3
Employment status		
Unemployed	46	80.7
Self-employed	9	15.8
Employed	2	3.5
Marital status		
Married	17	29.8
Unmarried	30	52.6
Divorced	10	17.5

### 3.2 Participants' therapy characteristics

The participants had been treated with antipsychotics for an extended period of time, 3 to 25 years. Table 4. presents prescriptions for both typical and atypical antipsychotics, whether prescribed alone or in combination. Most of the participants reported experiencing side effects while on antipsychotic treatment. Most participants experience side effects such as extrapyramidal symptoms, sedation, and metabolic problems.

**Table 4:** Participants' therapy characteristics.

Variable	Description
Antipsychotics	Typical
	Haloperidol
	Chlorpromazine
	Trifluoperazine
	Atypical
	Risperidone
	Olanzapine
	Quetiapine
	Klozapine
Number of drug items per prescription	Minimal 1 item
	Maximal 5 item
Duration of treatment	Minimal 3 years
	Maximal 25 years
Side effects	Extrapyramidal symptoms
	Dry mouth
	Constipation
	Sedation
	Sialorrhea
	Akathisia
	Tardive dyskinesia
	Weight gain

### 3.3 PANSS score

**Fig. 2:**
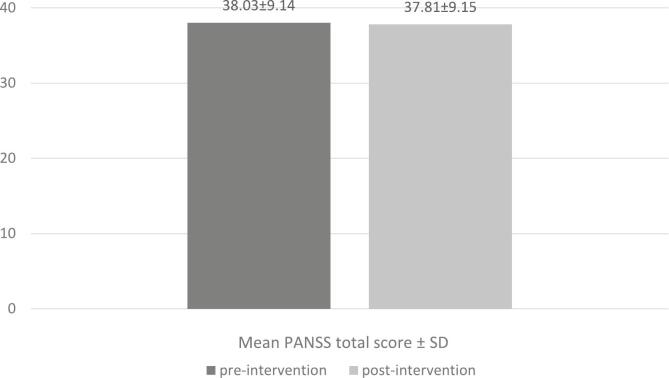
Comparison of Mean PANSS Total Scores Pre-and Post-Intervention.

As presented in Fig. 2, mean PANSS total scores showed a minimal decrease from 38.03 ± 9.14 at baseline (T0) to 37.81 ± 9.15 post-intervention (T4), a change that was not statistically significant (*P* = 0.089), indicating stability in symptoms rather than significant improvement.

### 3.4 Medication adherence

**Fig. 3:**
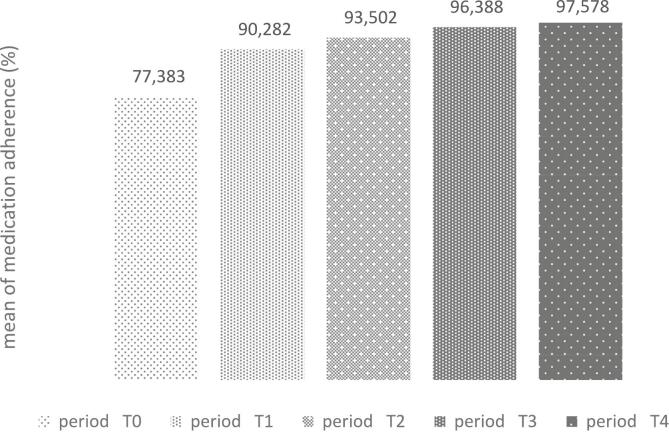
Improvement in Mean Medication Adherence from Baseline (T0) to Final Visit (T4) During Si-Care Intervention.

Table 5 and Fig. 3 show that mean medication adherence improved significantly from 77.38 % ± 25.85 at baseline (T0) to 97.57 % ± 11.09 at the final visit (T4).

**Table 5:** Adherence to medication of participants before and after intervention.

Period	Medication adherence	*P*-value
	(Mean ± SD)	
1st home visit (T0)	77.38 ± 25.85	0.000⁎
3th home visit (T1)	90.28 ± 16.98	
5th home visit (T2)	93.50 ± 15.47	
6th home visit (T3)	96.38 ± 12.79	
7th home visit (T4)	97.57 ± 11.09	

⁎ Friedman test.

## 4 Discussion

### 4.1 Characteristics of the participants

The profile of the participants in this study, including low educational attainment, high unemployment, and a predominantly unmarried status, reflects the socioeconomic challenges commonly observed among people with schizophrenia. These characteristics align with findings from other research, which highlight that people with schizophrenia often face significant barriers to educational and occupational achievement due to the early onset of symptoms, such as cognitive impairment and social withdrawal.^25^^,^^26^ Based on the results of several studies, the employment rate for people with schizophrenia is relatively low.^27^, ^28^, ^29^ Other barriers emerge after the initial episode, such as symptoms (including cognitive deficits), side effects of medications, and stigma, all of which can prevent patients from returning to education after experiencing a psychotic episode.^25^^,^^30^

The high percentage of unmarried participants (52.6 %) also aligns with literature that associates schizophrenia with a reduced likelihood of forming and maintaining intimate relationships. Most people with schizophrenia are not married due to several interrelated factors that stem from the nature of the illness and its impact on social functioning. Schizophrenia often manifests in early adulthood, a critical period for forming romantic relationships and entering into marriage.^31^ The early onset of symptoms, such as social withdrawal, emotional detachment, and cognitive impairment, can hinder people from engaging in meaningful interpersonal relationships. Furthermore, the stigma surrounding mental illness, particularly schizophrenia, further complicates the ability to form and maintain relationships, as potential partners can have misconceptions or fears about the condition.^32^

In addition, the functional impairments associated with schizophrenia, such as difficulties in maintaining employment, managing daily tasks, and dealing with the stress of social interactions, make it harder for people to meet the social and economic expectations often associated with marriage.^32^^,^^33^ The long-term need for antipsychotic treatment and the side effects of medications can also add to the challenges of maintaining a stable relationship, as these factors can strain emotional and physical intimacy.^34^ Consequently, people with schizophrenia face significant barriers to marriage, leading to a higher rate of singlehood compared to the general population.

### 4.2 Treatment characteristics of participants

Antipsychotic use is standard in the treatment of schizophrenia. Antipsychotic medications are necessary for individuals with schizophrenia to be succeed in rehabilitation programs. Therefore, it is crucial to initiate drug treatment early, particularly within five years of the first acute episode, as this is when most of the disease-related changes in the brain occur.^35^^,^^36^ After receiving treatment during the acute phase, patients should undergo maintenance therapy, which aims to increase socialization, improve self-care and mood, and prevent relapse.^37^^,^^38^ Studies have shown that patients receiving maintenance therapy have a lower incidence of relapse (18 %–32 %) compared to those not receiving such therapy (60 %–80 %).^39^^,^^40^ The current recommendation is to maintain antipsychotic therapy for at least 12 months following the remission of the initial psychotic episode.^41^

However, there are a range of side effects, primarily related to the use of antipsychotic medications, both typical (such as haloperidol and chlorpromazine) and atypical (such as risperidone and olanzapine). Commonly reported side effects included extrapyramidal symptoms (EPS), such as tremors, muscle stiffness, and tardive dyskinesia, which are common with typical antipsychotics. EPS can be very distressing and often lead to nonadherence in people with schizophrenia.^6^^,^^42^

According to the findings of a survey study, a large number of people with schizophrenia reported experiencing at least one side effect of their medication.^43^ These side effects can negatively affect medication adherence, as discomfort or fear of adverse effects often lead patients to stop taking their prescribed medications or take them irregularly, significantly affecting their quality of life.^43^^,^^44^ Therefore, understanding and addressing these side effects is critical to improving adherence and overall treatment results.

The study participants reported experiencing sedation, such as excessive drowsiness, especially with atypical antipsychotics like olanzapine and quetiapine. Sedation can disrupt daily functioning, making it difficult for patients to participate in routine activities, which can negatively impact adherence.^45^

Antipsychotic have anticholinergic effects and anticholinergic effects are also frequent, such as dry mouth and constipation, which are common side effects that impact patient comfort.^46^ These symptoms can lead to other problems, such as tooth decay and gastrointestinal obstruction.^45^ First-generation antipsychotics (FGA) with low potency and clozapine are highly likely to cause anticholinergic effects. Olanzapine and quetiapine have been shown to cause anticholinergic effects at high dosages.^47^

The last are metabolic side effects, such as weight gain, particularly associated with atypical antipsychotics, and these are common concerns with long-term antipsychotic use. Olanzapine and risperidone had the highest reported weight gain, olanzapine showing the most significant increase in BMI.^45^^,^^48^

One of the core components of the Si-Care intervention was counseling and education, where pharmacists played a critical role in helping patients and their families or caregivers manage and understand side effects. In this study, pharmacists offered strategies to deal with side effects, such as encouraging patients to communicate side effects with their doctors, which could lead to medication adjustments (e.g., changing doses or switching medications), educating patients about the importance of continuing medication despite side effects, while working to find solutions to manage those side effects, and providing psychoeducation to alleviate fear by explaining that side effects such as EPS or sedation can be managed and sometimes improve over time.

This educational support and individualized care were instrumental in maintaining and improving adherence. The pharmacists' regular follow-up through home visits created an opportunity to catch these issues early and address them before they led to nonadherence.

### 4.3 PANSS score

Higher total PANSS scores are linked to lower levels of adherence to treatment. This indicates that patients who display more severe symptoms, as indicated by their total PANSS score, are more likely to not adhere to their treatment. This finding aligns with another study, which revealed that patients with high PANSS scores exhibited poor compliance. Specifically, it noted that patients experiencing victimization delusions were likely to refuse medication and those with negative symptoms struggled with adherence due to a lack of motivation.^8^^,^^49^

PANSS (Positive and Negative Syndrome Scale) is a tool used to assess the severity of positive symptoms (such as hallucinations and delusions) and negative symptoms (such as apathy and loss of motivation) in patients with schizophrenia. The total PANSS score encompasses all these symptoms, so a higher score indicates more severe psychopathological conditions.^50^^,^^51^

Although the PANSS score did not show significant change, it is important to highlight that the participants' symptoms remained stable throughout the study period. This indicates that the Si-Care intervention effectively contributed to maintaining consistent symptom control. This stability is a crucial achievement in the context of schizophrenia, where nonadherence often leads to relapse and symptom exacerbation. By promoting adherence, the Si-Care intervention likely played a key role in preventing symptom relapse, a critical objective in the long-term management of schizophrenia.

There are several reasons why there was no significant decrease in PANSS scores. The study participants were stable outpatients who had received treatment for a long time, so their symptoms were more likely to be controlled, making it difficult to achieve further reductions in symptom severity. In addition, many participants had been living with schizophrenia for a long time, which is often associated with persistent symptoms, especially negative symptoms. These negative symptoms, such as social withdrawal, lack of motivation, are difficult to treat with medication.^52^ Although positive symptoms can be treated with antipsychotic drugs, negative symptoms are responsible for much of the long-term morbidity and functional impairment in patients with schizophrenia, so this will also affect the effectiveness of the Si-Care program on the overall PANSS score.^51^

The Si-Care intervention, which involved pharmacist-led home visits, counseling, and psychoeducation, likely played a significant role in maintaining symptom control by ensuring that patients adhered to their medication regimens and addressing common barriers to adherence, such as side effects and lack of understanding of the disease. Pharmacists consistently monitored the intake of medications in patients, provided customized support, and offered solutions for manage side effects, all of which are essential to maintaining symptom stability.

### 4.4 Adherence to medication

At the beginning of the study (T0), the mean adherence to medications among participants was 77.38 % ± 25.85. This baseline figure reflects the adherence challenges commonly seen in people with schizophrenia. Previous studies have shown that non-adherence rates among people with schizophrenia can range from 40 % to 60 %, due to various factors such as side effects of antipsychotic medications, lack of insight or understanding of the illness, forgetfulness or cognitive impairment, social stigma, and lack of support.^3^^,^^53^^,^^54^

Lack of knowledge or understanding of the disease was a factor that influenced people with schizophrenia. Often, they struggle with poor insight into their condition, which can lead to non-compliance as they may not recognize the need for ongoing medication.^55^ Schizophrenia is also frequently associated with cognitive deficits, making it difficult for patients to remember to take their medication.^56^^,^^57^ Another factors, patients may face societal stigma or lack of family support, leading to feelings of isolation or embarrassment about their treatment, further hindering adherence.^7^

The study showed a consistent and significant improvement in medication adherence after the Si-Care intervention, with adherence rates increasing at each time point (see Table 5). The mean medication adherence improved significantly from 77.38 % ± 25.85 at baseline (T0) to 97.57 % ± 11.09 at the final visit (T4), with the change statistically significant (Friedman test, *p* = 0.000).

This consistent increase underscores the success of the Si-Care intervention, especially the pharmacist-led education and support components, in overcoming barriers to adherence and encouraging patients to follow their medication regimen.

Improvement in adherence can be attributed to several key components of the Si-Care intervention. The core element of the Si-Care program was the involvement of pharmacists who conducted home visits and provided one-on-one counseling. In this study, pharmacists provided customized support to address specific issues that patients faced, such as managing side effects and psychoeducation. Pharmacists educated patients on how to manage these side effects and, where necessary, recommended medical consultations to adjust treatment. By alleviating these concerns, pharmacists helped patients feel more comfortable continuing their medication.

The Si-Care intervention effectively addressed several critical factors known to influence medication adherence in schizophrenia. Pharmacists played a central role in providing tailored education and counseling to patients and their families or caregivers.^58^^,^^59^ By educating patients about their illness, the importance of adherence, and the risks of non-compliance, pharmacists helped improve patients' understanding of why medication is essential for managing schizophrenia.^14^ Educational booklets also reinforced key concepts during home visits, ensuring that patients were consistently reminded of the importance of adherence. In line with previous research involving people with schizophrenia, a pragmatic pharmacy-based intervention demonstrated an improvement in antipsychotic adherence among this patient population.^14^^,^^60^

The pharmacists made seven home visits over four months, providing ongoing support and reinforcement of medication adherence strategies. These regular checks allowed the monitoring of adherence through pill counts and created a sense of accountability for the patients. The consistent presence of a healthcare professional who took a personal interest in the patient's progress probably contributed to their increased motivation to follow up treatment.^14^

One of the significant barriers to adherence in schizophrenia are the side effects associated with antipsychotic medications.^61^ In the Si-Care intervention, pharmacists identified and addressed side effects during their visits. By offering solutions such as consulting with the treating psychiatrist for dosage adjustments or recommending strategies to manage side effects, the intervention helped reduce the burden of these adverse effects and improve adherence.^12^

Another critical aspect of the Si-Care intervention was including family members and caregivers in education and counseling sessions. By educating families or caregivers about schizophrenia and their role in supporting medication adherence, the program strengthened the support system around the patient. Families or caregivers were better equipped to remind and encourage patients to take their medications, helping to maintain adherence over time.^62^

The sustained improvement in medication adherence seen in the Si-Care intervention has important implications for the long-term treatment of schizophrenia. Consistent adherence to medication is critical to prevent relapse and maintain symptom control in people with schizophrenia. Patients who adhere to their medication regimens are less likely to experience acute exacerbations of symptoms, reducing the risk of hospitalization and improving their overall quality of life.

Although the Si-Care intervention was successful in improving adherence, several limitations, challenges, and considerations for future interventions should be noted. First, the study used a quasi-experimental design without a control group, making it difficult to attribute the improvements in adherence to medication and symptom stability solely to the intervention. The absence of a comparison group means that external factors, such as prior treatment or natural progression of the disease, could have influenced the results.

However, in this study, the decision not to include a control group was influenced by ethical considerations. The pharmacist-led Si-Care intervention aims to improve medication adherence and symptom stability in patients with schizophrenia, who are often vulnerable to challenges in maintaining long-term adherence due to medication side effects, cognitive limitations, and social stigma. Providing a control group without the intervention risks increasing the likelihood of worsening their condition, as poor adherence in schizophrenia is strongly associated with relapse, hospitalization, and decreased quality of life.

Ethical principles such as beneficence and nonmaleficence suggest that withholding potentially beneficial interventions from vulnerable individuals may be inappropriate, especially when alternative designs such as quasi-experimental ones can still provide valuable insights.^63^ By not including a control group, this study sought to prioritize patient well-being, with all participants provided with support to improve adherence and symptom control, which is known to reduce the risk of relapse and improve clinical outcomes. This approach is consistent with similar intervention studies in schizophrenia, where ethical concerns about the no-intervention group have led to adjustments or removal of the control group to protect participant well-being, as non-adherent patients without additional support are at high risk of negative outcomes. While randomized controlled trials (RCTs) can provide stronger evidence, quasi-experimental designs offer a balanced approach, where health service development can still be pursued without compromising ethical standards.

Second, the study's sample size was relatively small, with only 57 participants, which may limit the generalizability of the findings to a broader population. Third, the study focused on a specific population of long-term outpatients who had been receiving antipsychotic treatment for several years. This limits the applicability of the findings to newly diagnosed patients or those experiencing acute phases of schizophrenia. The impact of the intervention on these groups may differ and requires further investigation.

Fourth, the study's four-month duration may not have been long enough to assess the sustainability of the improvements in medication adherence over time. Future research should consider longer follow-up periods to provide a better understanding of the long-term effects of this intervention.

Fifth, However, although considered objective, pill count method also has limitations, such as the possibility that patients throw away unused pills before counting, which can lead to higher estimates of adherence than actual. This method measures only the number of pills taken or left without ensuring that the drug is actually taken, especially for patients who may need to help understand the need for the drug.^64^^,^^65^ Furthermore, in Indonesia in particular, many individuals with schizophrenia depend on family members to manage their medication. If the caregiver does not monitor or record the amount of the drug consistently, this can affect the data on the number of pills. Another problem is limited access to health care, especially in rural and remote areas, where health visits and limited resources mean that patients may be limited to consistent services, such as restrictions due to distance and transportation to health care, leading to empty tablets and affecting the accuracy of pill counts. Although the pill count method is practical, it can provide a more comprehensive picture of medication compliance when combined with other adherence measurement methods.

Finally, The Si-Care intervention utilizes a relatively low-cost method (pill counting) that can be administered by a trained community pharmacist during a home visit. This makes it suitable for resource-constrained settings where sophisticated technology may be difficult to implement. However, because the intervention relies on repeated in-person pharmacist visits, scalability may be limited in areas with inadequate healthcare staffing or geographic challenges.To achieve scalability, the Si-Care program must be adaptable to the resources available in each location, including healthcare staff, costs, and infrastructure. For instance, if the program is implemented in rural areas or regions with limited healthcare access, alternatives such as telemedicine may be needed to reduce dependence on in-person pharmacist visits, allowing the program to run effectively regardless of the setting.

With these improvements, future studies could yield stronger and more generalizable results, significantly contributing to the development of effective, sustainable care models for schizophrenia. Additionally, future research could consider comprehensive evaluation methods, such as satisfaction surveys, in-depth interviews with patients and caregivers, and cost-benefit analyses. This in-depth evaluation would help identify the most effective elements of the program and areas needing adjustment, allowing it to evolve into a more sustainable and applicable model across various healthcare settings.

## 5 Conclusion

In summary, the Si-Care intervention proved effective in improving medication adherence, a crucial factor in schizophrenia management, and contributed to maintaining stable symptoms. The home-based model led by pharmacists provided valuable support for overcoming adherence challenges in schizophrenia. Despite the limitations, this study highlights the potential role of pharmacists in mental health care. Future research should consider a more rigorous design, a larger sample size, and longer follow-up to better evaluate the sustainability, scalability, and applicability of the intervention in diverse healthcare settings.

## Funding

This study was supported by Dana Abadi Perguruan Tinggi (DAPT) 2023, 10.13039/501100012521Universitas Gadjah Mada with Grant Number 1706/UN1/FA/SETPIM/KP/2024.

## CRediT authorship contribution statement

**Noor Cahaya:** Writing – review & editing, Writing – original draft, Methodology, Investigation, Formal analysis, Data curation, Conceptualization. **Susi Ari Kristina:** Writing – review & editing, Visualization, Validation, Supervision, Methodology, Conceptualization. **Anna Wahyuni Widayanti:** Writing – review & editing, Validation, Supervision, Methodology, Formal analysis, Conceptualization. **James A. Green:** Writing – review & editing, Validation, Supervision, Methodology, Formal analysis, Conceptualization.

## Declaration of competing interest

The authors declare that they have no known competing financial interests or personal relationships that could have appeared to influence the work reported in this paper.

## References

[1] Rasool S., Zafar M.Z., Ali Z., Erum A. (2018). Schizophrenia : an overview. Clin Pr.

[2] World Health Organization (2024). Schizophrenia Fact Sheets. [Internet]. https://www.who.int/news-room/fact-sheets/detail/schizophrenia.

[3] Mohammed F., Geda B., Yadeta T.A., Dessie Y. (2024). Antipsychotic medication non-adherence and factors associated among patients with schizophrenia in eastern Ethiopia. BMC Psychiatry.

[4] El Abdellati K., De Picker L., Morrens M. (2020). Antipsychotic treatment failure : a systematic review on risk factors and interventions for treatment adherence in psychosis. Syst Rev.

[5] Hiwot Sumesh, Henock A. (2018). A study to assess the prevalence of antipsychotics non-adherence and it’s associated factors among patients with schizophrenia in. JOJ Nurse Heal Care.

[6] Divac N., Prostran M., Jakovcevski I., Cerovac N. (2014). Second-generation antipsychotics and extrapyramidal adverse effects. Biomed Res Int.

[7] Meepring S., Tulyakul P., Sathagathonthun G., Supasri J. (2021). A review of factors relating to medication non-adherence in patients with schizophrenia. Global J Health Sci.

[8] Chaudari B., Saldanha D., Kadiani A., Shahani R. (2017). Evaluation of treatment adherence in outpatients with schizophrenia. Ind Psychiatry J.

[9] Saba N.U., Muraraiah S. (2022). Medication adherence and its associated factor: a cross-sectional study among patients with schizophrenia. Pharmacol Clin Pharm Res.

[10] Sendt K.V., Tracy D.K., Bhattacharyya S. (2015). A systematic review of factors influencing adherence to antipsychotic medication in schizophrenia-spectrum disorders. Psychiatry Res.

[11] Semahegn A., Torpey K., Manu A., Assefa N., Tesfaye G., Ankomah A. (2020). Psychotropic medication non-adherence and its associated factors among patients with major psychiatric disorders: a systematic review and meta-analysis. Syst Rev.

[12] Howe J., Lindsey L. (2023). The role of pharmacists in supporting service users to optimise antipsychotic medication. Int J Clin Pharm.

[13] Samprasit N., Bunchuailua W., Kapol N. (2020). Outcomes of pharmacist intervention in schizophrenic patients : a systematic review and Meta-analysis of randomized controlled trials. Srinagarind Med J.

[14] Mishra A. (2017). Impact of pharmacist-led collaborative patient education on medication adherence and quality of life of schizophrenia patients in a tertiary care setting. Bull Fac Pharm Cairo Univ.

[15] Sathienluckana T., Unaharassamee W., Suthisisang C., Suanchang O., Suansanae T. (2018). Anticholinergic discontinuation and cognitive functions in patients with schizophrenia : a pharmacist – physician collaboration in the outpatient department. Integr Pharm Res Pract.

[16] Barkhof E., Meijer C.J., De Sonneville L.M.J., Linszen D.H., De Haan L. (2013). The effect of motivational interviewing on medication adherence and hospitalization rates in nonadherent patients with multi-episode schizophrenia. Schizophr Bull.

[17] Ramadan A.A., Taha A.T., Madbouly N., Albasosi A.I. (2018). Effect of motivational interviewing- based intervention on medication attitude and adherence in patients with mental illness. J Nurs Heal Sci.

[18] Loots E., Goossens E., Vanwesemael T., Morrens M., Van Rompaey B., Dilles T. (2021). Interventions to improve medication adherence in patients with schizophrenia or bipolar disorders: a systematic review and meta-analysis. Int J Environ Res Public Health.

[19] Tan Y.M., Chong C.P., Cheah Y.C. (2019). Impact of hospital pharmacist-led home medication review program for people with schizophrenia : a prospective study from Malaysia. J Appl Pharm Sci.

[20] Ahamad T., Ananda K.N., Ghanashyam S.D., Bheemsain T.V. (2019). Effectiveness of clinical pharmacist led collaborative approach towards medication adherence in patients with schizophrenia receiving atypical antipsychotics at tertiary care hospital. Int J Res Ayurveda Pharm.

[21] Aburamadan H.A.R., Sridhar S.B., Tadross T.M. (2018). Intensive monitoring of adverse drug reactions to antiepileptic drugs in neurology Department of a Secondary Care Hospital in UAE. Int J Pharm Investig.

[22] Liechti S., Capodilupo G., Opler D.J., Opler M., Yang L.H. (2017). A developmental history of the positive and negative syndrome scale (PANSS). Innov Clin Neurosci.

[23] Lam W.Y., Fresco P. (2015). Medication adherence measures : an overview. Biomark Neuropsychiatry.

[24] Haddad P.M., Brain C., Scott J. (2014). Nonadherence with antipsychotic medication in schizophrenia: challenges and management strategies. Patient Relat Outcome Meas.

[25] Tesli M. (2022). Educational attainment and mortality in schizophrenia. Acta Psychiatr Scand.

[26] Dickson H. (2020). Academic achievement and schizophrenia: a systematic meta-analysis. Psychol Med.

[27] Yıldız M. (2019). Rates and correlates of employment in patients with schizophrenia: a multicenter study in Turkey. Int J Soc Psychiatry.

[28] Evensen S., Wisløff T., Lystad J.U., Bull H., Ueland T., Falkum E. (2016). Prevalence, employment rate, and cost of schizophrenia in a high-income welfare society: a population-based study using comprehensive health and welfare registers. Schizophr Bull.

[29] Erim B.R., Boztaş H., Yıldız M., Uygun E. (2019). The factors affecting the relationship between remission status and employment in chronic schizophrenia patients. Psychiatry Investig.

[30] Annapally S.R., Jagannathan A., Kishore T., Thirthalli J., Daliboina M., Channaveerachari N.K. (2019). Barriers to academic reintegration in students with severe mental disorders: thematic analysis. Asian J Psychiatr.

[31] Li X.J. (2015). The influence of marital status on the social dysfunction of schizophrenia patients in community. Int J Nurs Sci.

[32] Lyngdoh L.A., Antony S., Basavarajappa C., Kalyanasundaram J., Ammapattian T. (2023). Marriage in persons with severe mental illness: a narrative review-based framework for a supported relationship. J Family Med Prim Care.

[33] Nyer M. (2010). The relationship of marital status and clinical characteristics in middle-aged and older patients with schizophrenia and depressive symptoms. Ann Clin Psychiatry.

[34] Behere P.B. (2020). Is marriage solution for persons with schizophrenia?. Med Sci.

[35] Keating D. (2021). Prescribing pattern of antipsychotic episode psychosis : a medication for first- ­ retrospective cohort study. BMJ Open.

[36] Haddad P.M., Correll C.U. (2018). The acute efficacy of antipsychotics in schizophrenia : a review of recent meta-analyses. Ther Adv Psychopharmacol.

[37] Correll C.U., Rubio J.M., Kane J.M. (2018). What is the risk-benefit ratio of long-term antipsychotic treatment in people with schizophrenia ?. World Psychiatry.

[38] Patel K.R., Cherian J., Gohil K., Atkinson D. (2014). Schizophrenia: overview and treatment options. P T.

[39] Leucht S., Barnes T.R.E., Kissling W., Engel R.R., Correll C., Kane J.M. (2003). Relapse prevention in schizophrenia with new-generation antipsychotics: a systematic review and exploratory meta-analysis of randomized, controlled trials. Am J Psychiatry.

[40] Lehman A.F., Lieberman J.A., Dixon L.B., McGlashan T.H., Miller A.L., Perkins D.O. (2004). American Psychiatric Association practice guidelines; work group on schizophrenia. Practice guideline for the treatment of patients with schizophrenia. Am J Psychiatry.

[41] Tauscher-wisniewski S., Zipursky R.B. (2002). The role of maintenance pharmacotherapy in achieving recovery from a rst episode of schizophrenia. Int Rev Psychiatry.

[42] Peluso M.J., Lewis S.W., Barnes T.R.E., Jones P.B. (2012). Extrapyramidal motor side-effects of first-and second-generation antipsychotic drugs. Br J Psychiatry.

[43] DiBonaventura M., Gabriel S., Dupclay L., Gupta S., Kim E. (2012). A patient perspective of the impact of medication side effects on adherence: results of a cross-sectional nationwide survey of patients with schizophrenia. BMC Psychiatry.

[44] Ljungdalh P.M. (2017). Non-adherence to pharmacological treatment in schizophrenia and schizophrenia spectrum disorders - an updated systematic literature review. Eur J Psychiatry.

[45] Stroup T.S., Gray N. (2018). Management of common adverse effects of antipsychotic medications. World Psychiatry.

[46] Hilmer S.N., Gnjidic D. (2022). The anticholinergic burden: from research to practice. Aust Prescr.

[47] Solmi M. (2017). Safety, tolerability, and risks associated with first-and second-generation antipsychotics: a state-of-the-art clinical review. Ther Clin Risk Manag.

[48] Rognoni C., Bertolani A., Jommi C. (2021). Second-generation antipsychotic drugs for patients with schizophrenia: systematic literature review and meta-analysis of metabolic and cardiovascular side effects. Clin Drug Investig.

[49] Yu W. (2021). Analysis of medication adherence and its influencing factors in patients with schizophrenia in the Chinese institutional environment. Int J Environ Res Public Health.

[50] Opler M.G., Yavorsky C., Daniel D.G. (2017). Positive and negative syndrome scale (PANSS) training: challenges, solutions, and future directions. Innov Clin Neurosci.

[51] Leucht S. (2019). Linking PANSS negative symptom scores with the clinical global impressions scale: understanding negative symptom scores in schizophrenia. Neuropsychopharmacology.

[52] Correll C.U., Schooler N.R. (2020). Negative symptoms in schizophrenia: a review and clinical guide for recognition, assessment, and treatment. Neuropsychiatr Dis Treat.

[53] Phan S.V. (2016). Medication adherence in patients with schizophrenia. Int J Psychiatry Med.

[54] Misdrahi D. (2023). Predictors of medication adherence in a large 1-year prospective cohort of individuals with schizophrenia: insights from the multicentric FACE-SZ dataset. Transl Psychiatry.

[55] Kim J. (2020). Insight and medication adherence in schizophrenia: an analysis of the CATIE trial. Neuropharmacology.

[56] Settem V.J., Karanadi H., Praharaj S.K. (2019). Cognitive deficits, depressive symptoms, insight, and medication adherence in remitted patients with schizophrenia. Indian J Psychiatry.

[57] Uppinkudru C., Gopalakrishnan R., Noel J., Kuruvilla A. (2023). Prevalence, correlates and explanatory models of cognitive deficits in patients with schizophrenia - a cross sectional study. Indian J Psychiatry.

[58] Howe J., Lindsey L. (2023). The role of pharmacists in supporting service users to optimise antipsychotic medication. Int J Clin Pharm.

[59] Danladi J., Falang K.D., Barde R.A., Jimam N.S., Dangiwa D.A. (2013). Pharmaceutical care and medication adherence in management of psychosis in a Nigerian tertiary hospital. J Res Pharm Pract.

[60] Stuhec M., Bratović N., Mrhar A. (2019). Impact of clinical pharmacist’s interventions on pharmacotherapy management in elderly patients on polypharmacy with mental health problems including quality of life: a prospective non-randomized study. Sci Rep.

[61] Higashi K., Medic G., Littlewood K.J., Diez T., Granström O., de Hert M. (2013). Medication adherence in schizophrenia: factors influencing adherence and consequences of nonadherence, a systematic literature review. Ther Adv Psychopharmacol.

[62] Caqueo-urizar A., Rus-Calafell M., Urzua A., Escudero J., Gutiérrez-Maldonado J. (2015). The role of family therapy in the management of schizophrenia : challenges and solutions. Neuropsychiatr Dis Treat.

[63] Varkey B. (2021). Principles of clinical ethics and their application to practice. Med Princ Pract.

[64] Williams A.B., Amico K.R., Bova C., Womack J.A. (2013). A proposal for quality standards for measuring medication adherence in research. AIDS Behav.

[65] Nguyen T., La Caze A., Cottrell N. (2013). What are validated self-report adherence scales really measuring ?: a systematic review. Br J Clin Pharmacol.

